# Analysis of Thermomechanical Behavior of the Tubular Braided Fabrics with Flax/Polyamide Commingled Yarns

**DOI:** 10.3390/polym15030637

**Published:** 2023-01-26

**Authors:** Jinlei Li, Gildas L’Hostis, Nahiène Hamila, Peng Wang

**Affiliations:** 1 University of Haute-Alsace, Ensisa, Lpmt, F-68000 Mulhouse, France; 2 University of Strasbourg, F-67081 Strasbourg, France; 3 ENI Brest, IRDL, UMR CNRS 6027, F-29200 Brest, France

**Keywords:** natural fibers, tubular braided fabrics, thermoforming, thermomechanical behavior

## Abstract

Flax fibers are widely used as the strongest natural fibers in composite parts with advanced structures. Due to their excellent mechanical properties and recyclability, they have attracted more attention from the aviation and automotive industries, etc. These composite parts are usually obtained by preforming reinforcements or prepregs at high temperatures, and their mechanical behaviors are greatly affected by temperature variations. To improve the understanding of the mechanical properties of flax fiber materials, especially for the braided fabric with non-orthogonal structures, uniaxial tensile tests at different temperatures and tensile speeds were conducted on hollow tubular braided fabrics. The thermomechanical properties of Flax/Polyamide12 (PA12) prepregs were analyzed. The results show that temperature and tensile speed have obvious effects on the strength and shear stiffness of tubular fabrics. The strength and shear stiffness of the fabric decreases as the ambient temperature increases. Meanwhile, the strength of the fabric can also be improved by appropriately increasing the tensile speed. In addition, according to the experimental results, a theoretical model is established to describe the shear angle on the smallest circumference of the fabric, which provides a theoretical basis for the subsequent simulation process. The test results can provide a reference for the manufacture of flax fiber-reinforced composites with tubular structures.

## 1. Introduction

Textile-reinforced composites have attracted great interest in the aeronautic and automobile industries since they not only satisfy high-performance standards but also further reduce the overall weight of the structures [1,2]. In reducing the weight of complex components, textile composites exhibit significant potential. Natural fibers are found in abundance in nature and their properties are largely determined by their physical characteristics and chemical composition. Composites manufactured by natural fibers have superior mechanical, thermal and physicochemical properties [3]. Due to their low cost, recyclability and biodegradability, reinforcements manufactured from natural fibers like flax and cotton are gaining traction in textile composites. These characteristics allow them to reduce the mass of components while also saving costs and energy [4,5,6,7]. 

Generally, two textile processes are utilized to prepare textile reinforcements: weaving and braiding. The initial interlaced angle of the fabric prepared by the weaving process is 90° (the warp and weft are perpendicular to each other). The braiding process is suitable for the manufacture of flat or tubular fabrics. Due to the variability of the initial interlaced angle, the variety and style of the fabric made by the braiding process are more abundant than that of woven fabric. The inability to design interlaced angles for diverse shapes makes it difficult for woven reinforcements to satisfy real demand. Therefore, braiding has been gradually applied in the preparation of composite preforms [8]. Furthermore, the present paper focused on the studies of braided fabrics with non-orthogonal structures.

Thermoplastic prepregs with continuous fibers are usually performed in a thermoforming process, which is a frequent process to manufacture textile composite parts [4,5] due to the high productivity (typically 1 to 5 min) [9]. Temperature is one of the factors impacting the mechanical characteristics of materials during manufacturing, which has a direct influence on the forming quality of composite parts [10]. So, the thermomechanical behavior of the fabrics has been extensively analyzed. H. Lessard et al. [11] investigated the effect of different process parameters on component thickness, interlaminar shear strength and the degree of crystallinity during the thermostamping process. Thermomechanical behavior of woven carbon/polyphenylene sulphide and polyetheretherketone prepregs were studied by the bias-extension test [12]. It demonstrated that the final composite part is subject to temperature and shear behavior. Wrinkles are more easily induced, particularly when the process temperature is too low [12]. In addition, tensile and in-plane shear tests of Stretch Broken Carbon Fiber (SBCF) / Polyphenylene sulphide (PPS) and Polyetheretherketone (PEEK) commingled prepregs were conducted at different temperatures to analyze the thermomechanical properties [10]. In comparison to flax fiber, polyamide with the mixed properties of flax and PA12 can often be twined around the fiber yarn as reinforcements [13]. Xiao et al. [14] discussed the in-plane shear behavior of Flax /PA12 braided fabrics under the bias-extension test. The tensile behavior of a single yarn under different temperatures and speeds through a uniaxial tensile test was also studied. Simultaneously, the thermomechanical characteristics of woven fabrics relying on the bias-extension tests were also analyzed. It showed that the melting point of PA12 has a strong influence on tensile behavior [15]. Although many of the studies mentioned above have analyzed the thermomechanical properties of fabrics, they were all based on flat fabrics. In practical applications, the thermomechanical properties of tubular fabrics with higher structural integrity are less studied. Compared with flat fabrics, tubular fabrics can usually be used as reinforcement materials to be manufactured into medical catheters and sports equipment due to their special structural properties. 

As the most important deformation modes in the forming process, tensile and shear deformation have been extensively studied. These deformation modes determine the shape and mechanical properties of the final composite parts. Notably, the properties of the prepreg are not stable due to temperature variations during the thermoforming process. Therefore, the main objective of this paper is to identify the tensile and shear thermomechanical properties of tubular braided fabrics made by Flax/PA12 commingled yarns under varying temperatures and different speeds. It will further improve the understanding of the thermomechanical behavior of thermoplastic composites and facilitate effective numerical simulation of their forming processes to fully utilize the potential of the material to improve the quality of the part.

## 2. Materials and Methods

### 2.1. Materials

**Figure 1:**
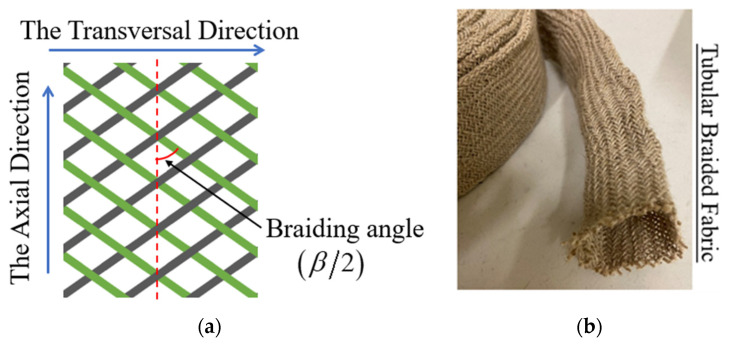
Tubular braided fabrics: (**a**) Structure diagram of 2-2 twill with braids and (**b**) Tested braids with Flax/PA12 commingled yarn.

The tubular braided reinforcements analyzed in the present study were manufactured from natural fibers commingled with Flax and Polyamide 12 (PA12) yarns (as shown in Figure 1b). The fabrics were produced by circular braiding loom (Herzog GLH 1/97/96-100) at Gemtex laboratory and described in Ref [8]. The total number of yarns in the tubular fabric is 96 and it was provided by the Schappe Technique. The length of the specimen used for the test is 40 mm excluding the gripper zones and the diameter of the specimen is 50 mm. The main physical properties are noted in Table 1. The braiding angle, one of the important parameters of the samples, is determined by the braiding process. It can be noted that half of the angle between the tows is defined as the braiding angle (β/2) which is 55°; the structure is shown in Figure 1a.

### 2.2. Tensile Tests under Hot Temperatures

**Figure 2:**
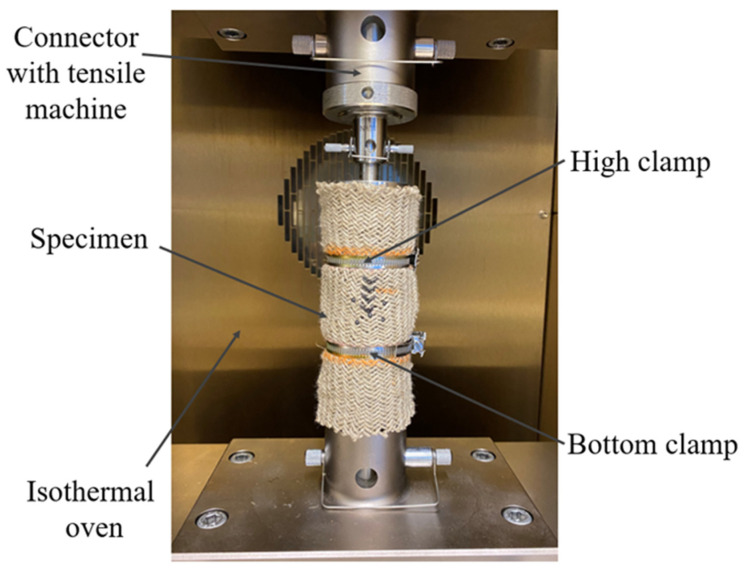
Setup of the tensile machine with the specimen.

The uniaxial tensile tests were conducted by an Instron tensile machine with a load sensor of 250 kN. According to the standard NF ISO 13934 [16], the tensile speed was set to 10 mm/min. To avoid the sliding of the sample during the stretching process, the ends of the fabric were connected to the stretching machine by the corresponding clamps. The mechanical behavior and the braiding angle of the samples during tensile tests were recorded thanks to a camera that was positioned in front of the machine. The software ImageJ was also used to analyze the pictures. Figure 2 displays the experimental setup. The thermomechanical test of the fabric is mainly divided into two parts to analyze the effects of temperature and tensile speed on mechanical properties. Since the melting value of PA12 is 178 °C, the temperature range was chosen to include values below and above the melt temperature to analyze the thermomechanical properties of fabrics more comprehensively. The specific experimental conditions are shown in Table 2. All tests were performed in an isothermal oven and each test was started after the temperature was stabilized. To ensure the accuracy of the results, the tests were conducted more than three times.

**Figure 3:**
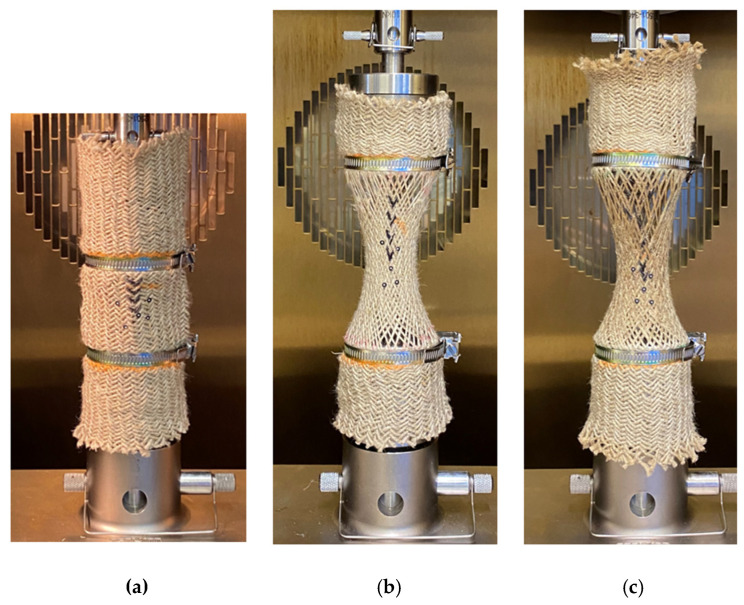
Tensile tests performed on tubular braids in an isothermal oven: (**a**) initial state; (**b**) specimen with broken yarn at 150 °C; and (**c**) specimen with broken yarn at 190 °C.

Figure 3 shows the tubular specimen before and after the tensile test. Figure 3a shows the initial state of the specimen. The deformed shape at 150 °C is shown in Figure 3b. Figure 3c shows the deformation at 190 °C. The radial shrinkage structure of the specimens is clearly shown in the deformed configuration. The melting state of PA12 can be observed at 190 °C and the specimen was impregnated.

The experimental results in Figure 3 show that the shear behavior of tubular fabric under tensile load not only leads to radial shrinkage but also to the yarns’ reorientation [17,18], which creates the shear angle. It is similar to the in-plane shear of the woven fabric [19,20,21]. During the bias-extension test, as the tensile displacement increased, the intersecting yarns rotated with each other and led to a decrease in the angle between them. So, the fabric undergoes in-plane shear behavior and produces the shear angle. The in-plane shear behavior leads to three different zones, which are the ‘no-shear zone’, ‘semi-shear zone’ and ‘pure shear zone’ [22,23,24]. It is worth noting that the shear angles are equal within the same zone. However, for tubular fabrics, the shear angle is the same only at the fabric’s circumference of the same height. To analyze the mechanical behavior of tubular fabrics, it is crucial to grasp the variation law of the shear angle; the shear angle not only depends on the tensile displacement but also is affected by the temperature. Therefore, to analyze the shear behavior of fabrics during thermoforming, the effects of deformation and temperature need to be considered. In the existing studies, the analysis of the shear angle is mostly based on analytical models which have proven to be effective for characterizing the mechanical behavior of fabrics at room temperature [19,22,25,26,27,28,29] and have been used for simulation analysis. This section attempts to develop an analytical model to determine the shear angle on the smallest circumference of the tubular fabrics at room temperature.

**Figure 4:**
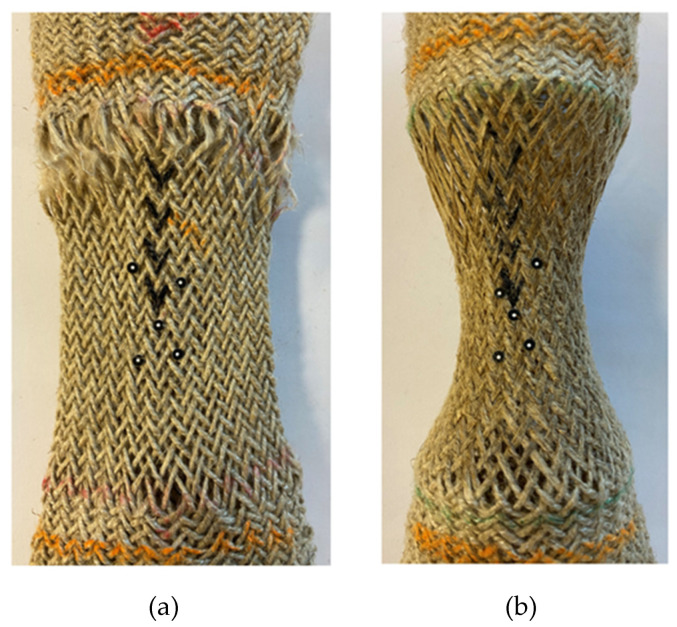
Specimens removed from the testing machine: (**a**) specimen at 150 °C and (**b**) specimen at 190 °C.

The state of the fabric after removing it from the tensile machine at 150 °C and 190 °C shows the results are different (Figure 4). It can be directly observed that the fabric still maintained the radial shrinkage shape even if it has been removed from the tensile machine after being stretched at the temperature of 190 °C. It is probably due to the fact that 190 °C exceeds the melting point of PA12; the fabric is in a molten state and cannot return to its initial state after curing. On the contrary, the melting point of PA12 is not reached at 150 °C and the fabric has the behavior of returning to its original state when it is removed from the machine. This also confirms that temperature has an important influence on the forming of the fabric and should be set reasonably. In particular, the melting point of the yarn cannot be ignored during the thermoforming process.

In the forming process of the fabric, the influence of the shear angle cannot be ignored. The shear angle facilitates the draping of the fabric but is also closely related to wrinkling. When the shear angle exceeds the maximum critical value (“locking angle”), wrinkling is more likely to be produced. So, it is necessary to master the evolution of the shear angle. It is obvious from the experimental results that the distribution of the shear angle of the tubular fabric is not uniform. The shear angle is largest in the middle part of the fabric and smallest at both ends. Therefore, the shear angle in the minimum circumference of the fabric (“the maximum shear zone”) is the first to reach the locking angle, which induces forming defects. In this section, an attempt is made to develop an analytical model to predict the shear angle in the maximum shear zone of tubular fabrics.

**Figure 5:**
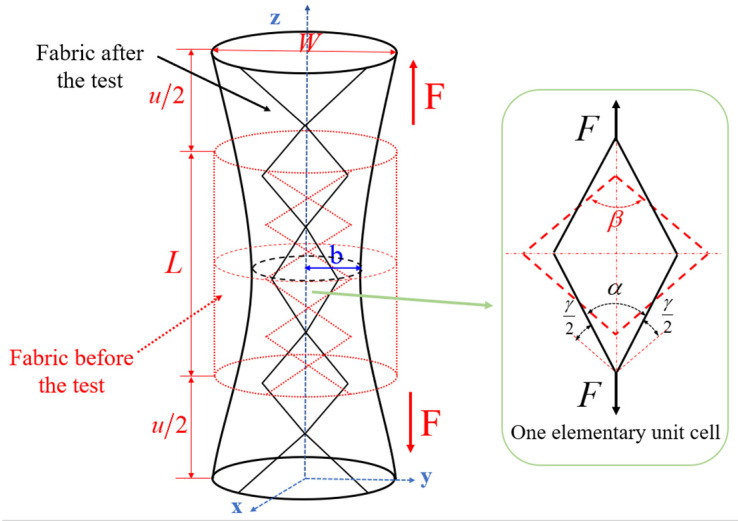
Deformation of the tubular fabric before and after the test.

According to the experimental results, a deformation schematic diagram of tubular fabric is shown in Figure 5. *L* and *W* in the figures symbolize the original length of the fabric and the diameter of the fabric, respectively. *z* represents the axial direction of the fabric. *F* is the tensile load. It is assumed that there is no slippage between two yarns and the yarns are not elongated during the test. As the tensile displacement increases, the relationship between the displacement and the minimum radius of the fabric can be fitted:

(1) $$b=0.005587\cdot u^{2}-0.5367\cdot u+25.06$$

where *u* presents the tensile displacement and *b* presents the minimum radius of the fabric.

To study the shear angle of the fabric, a unit cell in the minimum circumference of the fabric is taken as the research object, as shown in Figure 5. According to the definition of the shear angle and the corresponding geometric relationship, the shear angle on the minimum circumference of the fabric can be expressed as:

(2) γ=β−α

(3) $$\gamma =\beta -2\arcsin\left(\frac{2b\sin(\frac{\beta }{2})}{W}\right)$$

where *W* represents the diameter of the fabric.β is the initial interlaced angle and γ is the shear angle.

## 3. Results and Discussion

### 3.1. Comparison between the Experimental and Theoretical Shear Angle

**Figure 6:**
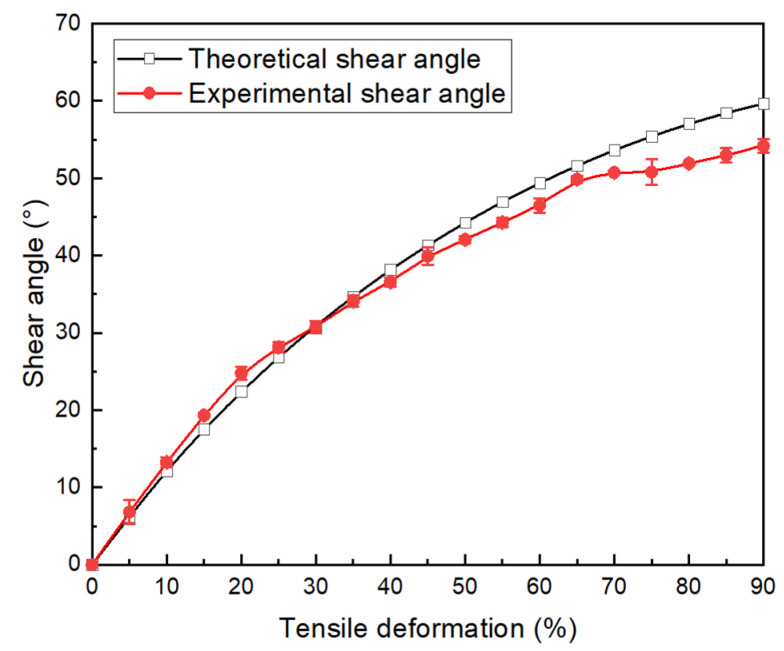
Comparison of the experimental and theoretical shear angle in the maximum shear zone of fabrics.

The shear angle in the minimum circumference of the fabric during the test was measured optically to verify the theoretical model proposed in Section 2. The theoretical value of the shear angle can be obtained by bringing the displacement into Equation (3) and compared with the experimental results, as shown in Figure 6. The measurement results of the shear angle in the minimum circumference of the fabric follow the theoretical curve until the deformation reaches 60% to 70% (corresponding to a 50° shear angle). When the fabric deformation reaches about 35% to 40% (i.e., a 35° angle), the experimental value and the theoretical value begin to separate, but the theoretical value can still describe the experimental value well. The maximum error at this stage is 9.5%. This means that there is no (or limited) elongation of the yarn and no (or limited) slippage between the two yarns before the 40% deformation, which is consistent with the previous assumptions. In this stage of the test, it is reasonable to use theoretical values to reflect the shear angle. The crossed yarns are rotated relative to each other, and the shear angle increases. When the deformation exceeds 70%, there is a large separation between the experimental curve and the theoretical curve. The maximum error at this stage is more than 10.4%. At the same time, the growth rate of the measured shear angle slows down, while the theoretical shear angle continues to increase. This can be due to slippage between the two yarns or yarn elongation. However, theoretical models do not take these two factors into account and thus cannot continue to describe the shear behavior of fabrics.

### 3.2. Comparison of Tensile Behavior of Flat Braided Fabric and Tubular Braided Fabric

**Figure 7:**
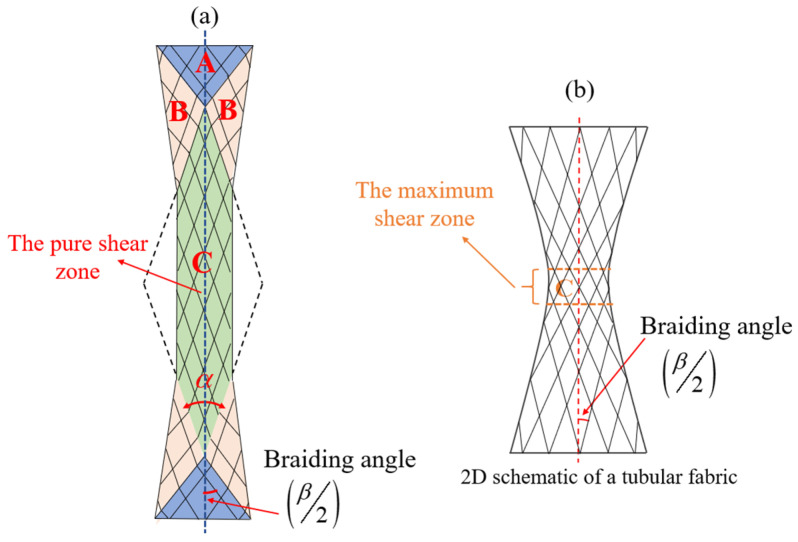
Schematic diagram of deformation of flat braided fabric (**a**) and tubular braided fabric (**b**).

The mechanical behavior of flat braided fabrics depends on the bias-extension test. The details of the test with Flax/PA12 flat braided fabrics are given in the literature [14] and fabrics with an aspect ratio of 4.2 were selected to compare with tubular fabrics; the tensile schematic is shown in Figure 7. Three distinct zones A, B and C are clearly indicated in Figure 7a. There is no shearing deformation in zone A, half shearing deformation in zone B and pure shearing deformation in zone C. The sample is stretched to produce the pure in-plane shear angle γ in zone C and semi-angle γ/2 in zone B. Compared with the stretching of flat fabrics, the deformation of tubular fabrics is more complicated. In the tensile test, the tubular fabric exhibits multiple different zones along its axial direction, and the shear angles of each zone are not equal. The distribution of the shear angle has a non-uniformity, the shear angle is largest near the middle part of the fabric and smallest near the ends. The shear angles are equal only if they are located on the same circumference of the fabric. At the same time, the maximum shear angle of flat fabric is in the pure shear zone, while the maximum shear angle of the tubular fabric is located on the minimum circumference of the fabric.

**Figure 8:**
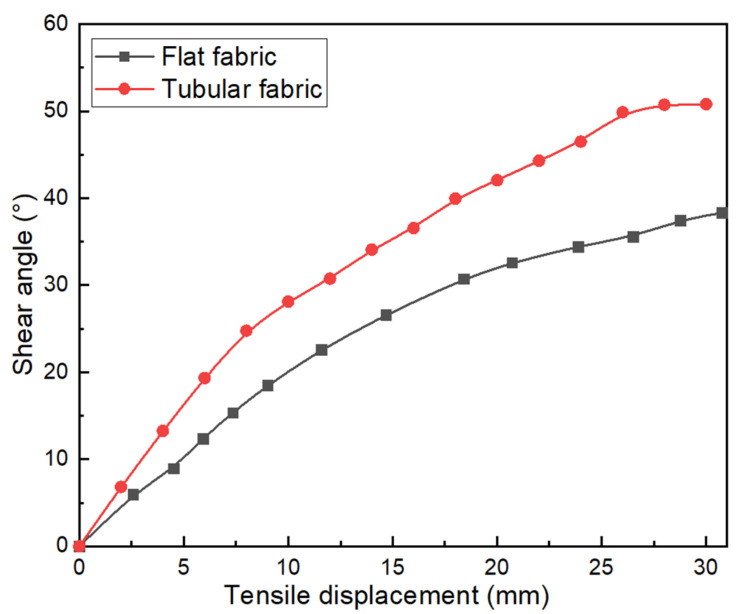
Comparison of the shear angle for flat braided fabric in the pure shear zone and tubular braided fabric in the maximum shear zone.

The braiding angle, one of the most important parameters of braided preform, has a great influence on the mechanical properties of the braided composite materials. The mechanical properties of braided preforms vary with the braiding angle. Therefore, the shear angle in the maximum shear zone of the flat braided fabric in the literature and tubular braided fabric in the present paper is compared during the stretching process, as shown in Figure 8. The maximum shear angle of the two fabrics increases first and then tends to be constant with the increase of tensile displacement. Under the same tensile displacement, the shear angle of the tubular fabric changes faster than that of the flat fabric. This is mainly because the middle zone of the tubular fabric has a larger shear space.

### 3.3. Characterizations of Tubular Braided Fabrics with Flax/PA12 Yarn

**Figure 9:**
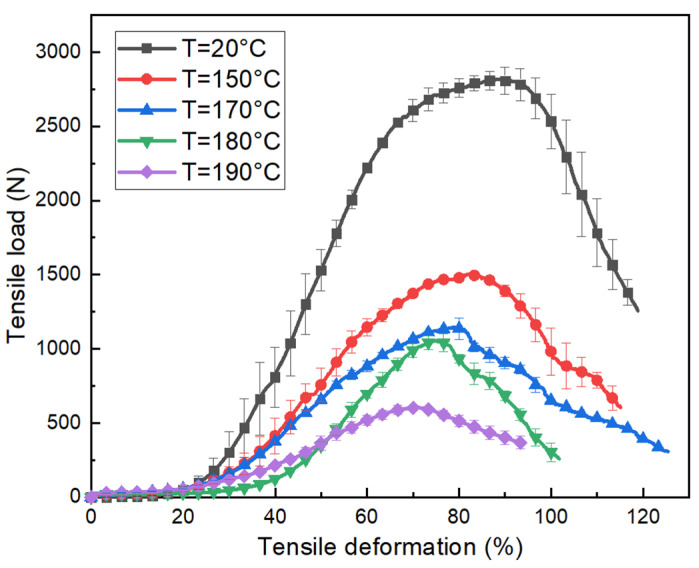
The tensile load vs. tensile deformation of tubular fabrics under variation of temperature.

The load-displacement curves of the tubular fabric at different temperatures are shown in Figure 9. The tensile curves of the fabric at different temperatures can be divided into three stages. In the first stage, the load increases relatively slowly with the increase of deformation. The load at this stage is necessary to overcome the adhesive friction between the yarns so that the yarns rotate with each other and start shearing until a deformation of about 35%~40%, which is consistent with the separation of the theoretical and experimental values of the shear angle mentioned in Figure 6. Similar to the bias-extension test, the fabric at this stage exhibits pure shear behavior. Then, the yarns contact each other and generate transversal compaction resulting in a rapid increase in load with the deformation increases. It is further explained that the growth rate of the in-plane shear angle slows down after the theoretical and experimental curves separate clearly. Finally, the load decreases due to the breakage of the fabric.

**Figure 10:**
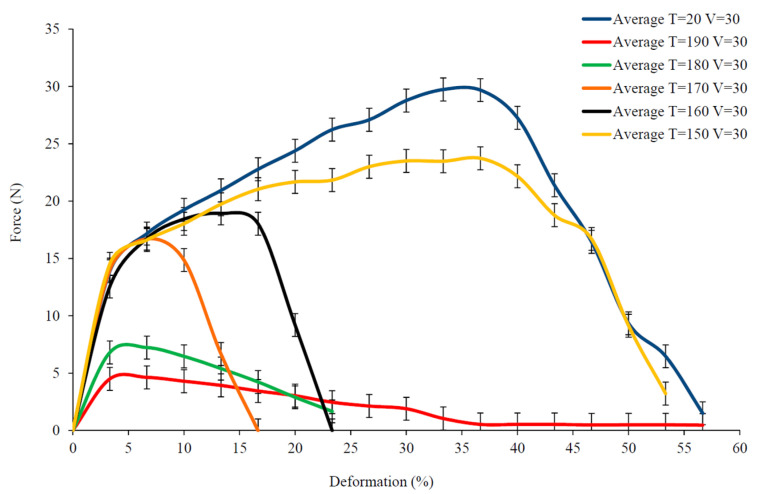
The tensile load vs. tensile deformation of single flax/PA12 yarn under variation of temperature.

Although all of the curves in Figure 9 present a similar non-linear evolution, the maximum load decreases with increasing temperature. The maximum load on the specimen at room temperature (T = 20 °C) is much larger than that at higher temperatures. In addition, the maximum deformation of the fabric also decreases with the temperature increases, and the fabric at 20 °C has a greater tensile deformation compared to 190 °C, which is strongly related to the melting point of PA12. The literature [15] gives the tensile results of single Flax/PA12 yarns at different temperatures, as shown in Figure 10.

The tensile behavior of a single yarn at room temperature is analyzed, specifically showing a large deformation as a relatively large force, which can be divided into three stages: rapid increase (<3%), slow increase (3–35%) and rapid decrease (>35%). The yarn in the first phase presents a shorter deformation corresponding to a large increase in tensile load. When the deformation reaches only about 3%, the slope of the curve changes, and the pure flax was broken; this has been proved by literature [30]. After the breakage of pure flax, PA12 was slightly deformed without breaking and it would still bear the load. The yarn retained a certain strength at this stage, the tensile process continues to the next phase. In the second stage, the larger deformation of yarn could be observed but the slope of the increasing load is slower. At this phase, pure flax was broken, PA12 exhibited tensile behavior and the yarn presents a progressive deformation until PA12 was broken. The curve enters the third stage after a second slope change. In the third stage, the load decreases sharply, indicating an increased progression of a slip of fracture in flax and PA12.

From the above discussion, in Figure 10, the tensile process of a single yarn at room temperature can be summarized as (1) pure flax breakage; (2) PA12 breakage; (3) slippage of flax and PA12. Comparing the tensile behavior of the yarn at other temperatures, it was found that as the temperature increased, the tensile behavior of the yarn gradually changed from the initial three parts to two parts, and the maximum deformation also decreased from the initial 35% to 3 %. When the temperature was lower than the melting temperature (178 °C) of PA12, the tensile curves still included the three stages. However, the deformation in the second stage decreased significantly as the temperature increased. This is because temperatures affect the strength of PA12, causing the force required to break PA12 to decrease as the temperature approaches its melting value. Therefore, compared with room temperature, the deformation before PA12 reached fracture also decreased with the increasing temperature. On the contrary, when the temperature was higher than the melting point of PA12, PA12 melted, and the tensile behavior of the yarn was mainly characterized by pure flax and the curve consisted of only two stages. The maximum load drops sharply. This is also the reason why the maximum load and maximum deformation of tubular fabric as shown in Figure 9 decrease with the increase in temperature during the stretching process. So, the thermomechanical behavior of the fabric depends largely on the temperature.

**Figure 11:**
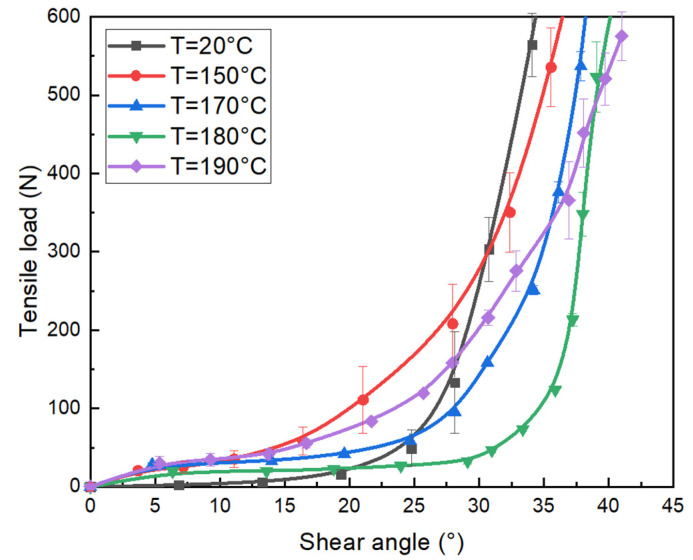
The tensile load vs. shear angle under the different temperatures of tubular fabrics.

The shear angle is an important parameter to reflect the mechanical properties of the specimen. The relationship between the shear angle and the load at different temperatures is shown in Figure 11. It can be found that at different temperatures, the shear angle and load present a non-linear evolution. Following the load augmentation, the shear angle increases. The increase in load can be divided into two stages. At first, the shear angle increases with a smaller load, then the shear angle becomes larger, and the load also increases noticeably. It is due to the pure shear behavior of fabric at the initial stage of the tensile test and the transverse compaction of the yarns after “the locking angle”. Similarly, the specimen at room temperature leads to load on the specimen that is much larger than for higher temperatures. This indicates that the shear stiffness of the fabric decreases with the increasing temperature. When the state of the fabric tends to be stable, as the test temperature increases, the shear angle increases under the same load conditions. This is also caused by the melt state.

**Figure 12:**
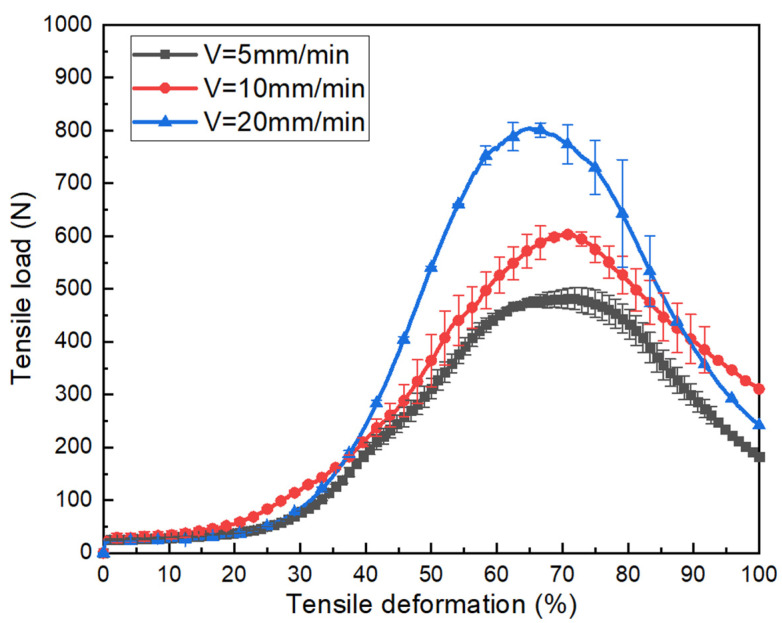
The tensile load vs. tensile deformation curves under the different tensile speeds of tubular fabrics at 190 °C.

The load versus deformation curves are plotted in Figure 12 corresponding to three different displacement rates. The temperature is maintained at 190 °C. Firstly, the thermomechanical properties of the fabric show similar evolution for the different tensile speeds, which are also divided into three phases. Secondly, it can be found that the maximum load of the fabric at a speed of 20 mm/min increases noticeably compared with the maximum load at a speed of 10 mm/min and 5 mm/min. This indicates that the displacement rate has a certain influence on the properties of tensile behavior. The shear stiffness improved by increasing the tensile speed. Under faster speed conditions, PA12 does not melt enough and the fabric maintains a high level of tensile resistance.

**Figure 13:**
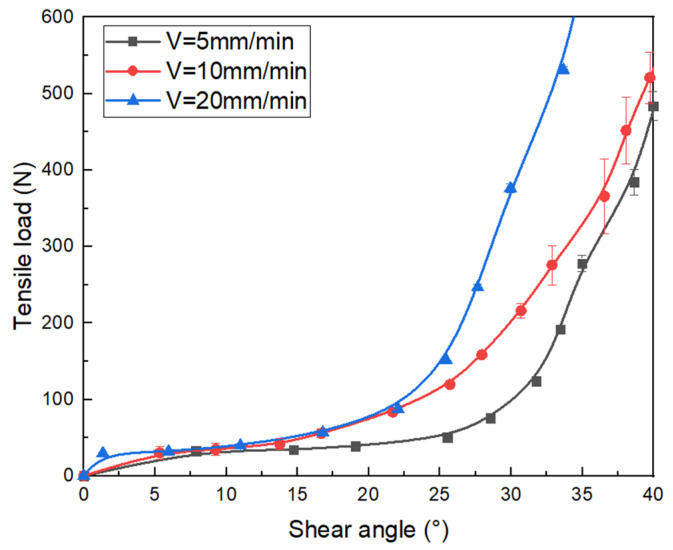
The tensile load vs. shear angle under variation of speed for tubular fabrics at 190 °C.

The shear angle characteristics corresponding to different tensile speeds of the fabric at 190 °C are shown in Figure 13. It can be observed that the fabric requires bigger effort at 20 mm/min than at 10 mm /min and 5 mm/min to achieve the same shear angle. Thus, this also proves that the strength of the fabric is influenced by the variation of the tensile speed. The strength of the fabric can be kept high by properly increasing the tensile speed.

## 4. Conclusions

In this paper, the thermomechanical properties of 2 × 2 Twill braided Flax/PA12 tubular fabric were characterized at different forming temperatures and tensile speeds. The results show that temperature and tensile speed affect the mechanical properties of the fabric. Compared with the curves at different temperatures, the fabric at room temperature has the highest shear stiffness. As the temperature increases, the state of the yarn gradually transforms from a solid state at room temperature to a liquid state above the melt temperature. The effect of lubrication reduces the friction between the yarns, so the shear stiffness is reduced and the maximum load gradually decreases.

The effect of tensile speed on the mechanical properties of the fabric is specifically shown by the fact that the strength of the fabric increases as the tensile speed increases. In the case of increasing the tensile speed, the fabric requires a bigger effort to achieve the same shear angle and the shear stiffness increases. In addition, the analytical model of shear angle is established in this paper to provide ideas for future numerical simulations to optimize the feasible condition in the manufacturing processes of thermoplastic composites. Since the bending stiffness of the fabric is closely related to wrinkling, to improve the forming quality of the composite parts, research on the bending properties of Flax/PA12 is very important and necessary.

## Figures and Tables

**Table 1:** The main properties of the tubular braid fabrics.

Parameters	Flax/PA12 Specimen
Type of braid	Biaxial twill 2-2
Initial braid angle (β/2)	55°
Yarns density (tex)	500
Area density (g/m^2^)	376 ± 5
Number of yarns per cm	4.2
Thickness (mm)	2.06
The mass fraction of flax	64%
The mass fraction of polyamide	36%

**Table 2:** The different conditions of the tensile test.

Title 1	Temperature (°C)	Velocity (mm/min)
Room temperature and displacement speed	20	10
Variants of temperature with same velocity	150	10
	170	
	180	
	190	
Variants of velocity with same temperature	190	5
		10
		20
